# Interpersonal synchrony feels good but impedes self-regulation of affect

**DOI:** 10.1038/s41598-019-50960-0

**Published:** 2019-10-11

**Authors:** Laura Galbusera, Michael T. M. Finn, Wolfgang Tschacher, Miriam Kyselo

**Affiliations:** 1 Inter-Self Lab, Institute of Philosophy, History of Literature, Science and Technology, Technische Universität Berlin, Berlin, Germany; 2 Brandenburg Medical School, Department of Psychiatry and Psychotherapy, Immanuel Klinik Rüdersdorf, Rüdersdorf, Germany; 3 Osher Center for Integrative Medicine, Vanderbilt University Medical Center, Nashville, TN USA; 4 University Hospital of Psychiatry and Psychotherapy, University of Bern, Bern, Switzerland

**Keywords:** Cooperation, Human behaviour

## Abstract

The social benefits of interpersonal synchrony are widely recognized. Yet, little is known about its impact on the self. According to enactive cognitive science, the human self for its stability and regulation needs to balance social attunement with disengagement from others. Too much interpersonal synchrony is considered detrimental for a person’s ability to self-regulate. In this study, 66 adults took part in the Body-Conversation Task (BCT), a dyadic movement task promoting spontaneous social interaction. Using whole-body behavioural imaging, we investigated the simultaneous impact of interpersonal synchrony (between persons) and intrapersonal synchrony (within a person) on positive affect and self-regulation of affect. We hypothesized that interpersonal synchrony’s known tendency to increase positive affect would have a trade-off, decreasing a person’s ability to self-regulate affect. Interpersonal synchrony predicted an increase in positive affect. Consistent with our hypothesis, it simultaneously predicted a weakening in self-regulation of affect. Intrapersonal synchrony, however, tended to oppose these effects. Our findings challenge the widespread belief that harmony with others has only beneficial effects, pointing to the need to better understand the impact of interaction dynamics on the stability and regulation of the human self.

## Introduction

Humans are no islands, but interconnected social beings. We continuously engage in social interactions and are affected by them in return. Important dimensions of this reciprocal process remain beyond our awareness. They play out as we automatically attune and synchronize our movements and actions with others^1–3^. Interpersonal synchrony is beneficial as it functions as a social glue^4^ and facilitates harmonious interactions between people^5–7^. Considerable evidence shows that synchrony improves cooperative and other social skills^8,9^.

However, an important question still remains: what is the impact of interpersonal synchrony on the self? In this study we draw on a recent hypothesis in enactive cognitive science, according to which the stability of the self vitally relies on a balance between attunement with others, and the need for independence from them^10,11^. Following this, we challenge the widespread assumption that being in harmony with others is unquestionably beneficial or desirable. We hypothesize that too much social attunement is detrimental to the self’s stability and regulatory processes. While having obvious relevance for our social abilities, interpersonal synchrony might thus actually have a negative trade-off when it comes to the self.

Enactive approaches to cognitive science have recently challenged an individualist notion of the human self, according to which there is a clear-cut distinction between an agent and the objectively-given material or social world^12,13^. Instead, the relation between agent and world is described in terms of non-linear interactive dynamics, whereby agent and environment are mutually constrained by each other^13,14^. On this dynamical view, the boundary of the agent is not given, but continuously “enacted” (i.e. brought forth) through a process of self-organization, in which the agent actively structures the exchanges with the environment and thereby generates and maintains a form of systemic stability^12^. Such self-organizing processes can be found at several levels of the individual organism, e.g. in metabolic and neurobiological homeostasis^15^, sensorimotor integration^13,16^, as well as in inter-limb coordination^17,18^. But they also occur beyond the individual organism, at the social or collective level^19^.

When several agents interact they can co-create new higher order forms of social self-organization, with properties irreducible to the participating individuals^19–21^. We see this in the spontaneous coordination of body movement^18,22^, for instance when two persons unintendedly synchronize their rocking chairs^23^, or in the interpersonal dynamics of joint dance and music improvisation^24^. Research on interpersonal synchrony has shown consistently that being entrained in interactive self-organizing dynamics has important advantages for social interactions and social skills^8,9^. Interpersonal synchrony strengthens the relation between persons^6,25–27^. It enhances empathy and prosocial behavior^8,9,28,29^, social affiliation^5,8,30^, cooperation^31–34^ as well as social cognition^8,35,36^.

To date, there has been little consideration for how being entrained in such higher-level social dynamics might impact the self-organizing processes of the individual agents. Interactive and individual self-organization have been studied mainly independently from another. Self-organization in the individual has for instance been observed behaviourally in the synchronization of movement between segments of the agent’s body^17^. Yet research on interpersonal synchrony has neglected these individual forms of bodily self-organization and has mostly only focused on the behavioural properties of the dyad. One reason for this might have been methodological limitations in being able to measure the whole body moving in an open-ended and multivariate fashion. Only recently, technological and methodological advances have introduced more complex and multivariate measurement of movement, which allow the simultaneous study of intra-bodily and inter-bodily self-organization dynamics (see e.g.^37–39^).

The failure to account for the interdependence of interactive and individual self-organization also applies to important psychological dimensions of the individual agent. Psychological processes pertaining to the agent’s self-organization can be broadly conceived as a person’s psychological ability to regulate herself, e.g. her sensations, needs, and affects. Compared to the extensive amount of evidence that exists for the social impact of synchrony, its effect on the individual’s psychological states and processes has received little attention. To our knowledge, research in this direction has mainly focused on the effect of interpersonal synchrony on a person’s affective states and found that it increases positive affect^8,9,40^. A few studies have furthermore shown that synchrony makes us feel more similar to and dependent on others^9,41^ and can also lead to experiences of self-other merging^42,43^. The weakening of self-other boundary associated to synchronization phenomena also yields less sensitivity to own bodily pain^44,45^ and higher empathy for the other’s pain^46,47^. Being in sync thus tends to feel good and creates a sense of connection in the individual agents. Yet importantly, its impact on the agent’s self-regulation remains under-investigated. Especially the findings that associate synchrony with the weakening of the self-other boundary point to the need to investigate how interpersonal synchrony impacts our ability to regulate psychological states, which is vital for maintaining individual self-organization.

A possibly disrupting impact of social attunement on individual self-regulation has been suggested by a recently formulated hypothesis in enactive cognitive science: the human self, for its stability and regulation, relies on both, individual (e.g. bodily) and interactional self-organizational loops^10,13^. Importantly however, in order to maintain a stable self, agents need to structure their entrainment in social interaction dynamics so as to also allow for moments of independence and disengagement. The human self is, at its core, neither completely attuned to nor separated from the social environment but integrates both dimensions instead^10^. Not balancing them can have negative effects. As research on solitary confinement showed, a lack of social engagements can have severe consequences for a person’s sense of self and regulation capacities^48,49^. But by the same token, an excess of social attunement and synchrony might lead to dysregulation and instability of the self, as well^50^.

For the present study we thus assumed that interpersonal synchrony matters not only with regards to our social interactions or social skills but also to individual self-organization. We departed from previous research, which has mainly looked at the social impact of interpersonal synchrony, and focused instead on its impact on the self. This shift of focus also required important technological and methodological advances to facilitate the measurement of spontaneous movement dynamics occurring both *between* selves and *within* the self simultaneously.

**Figure 1:**
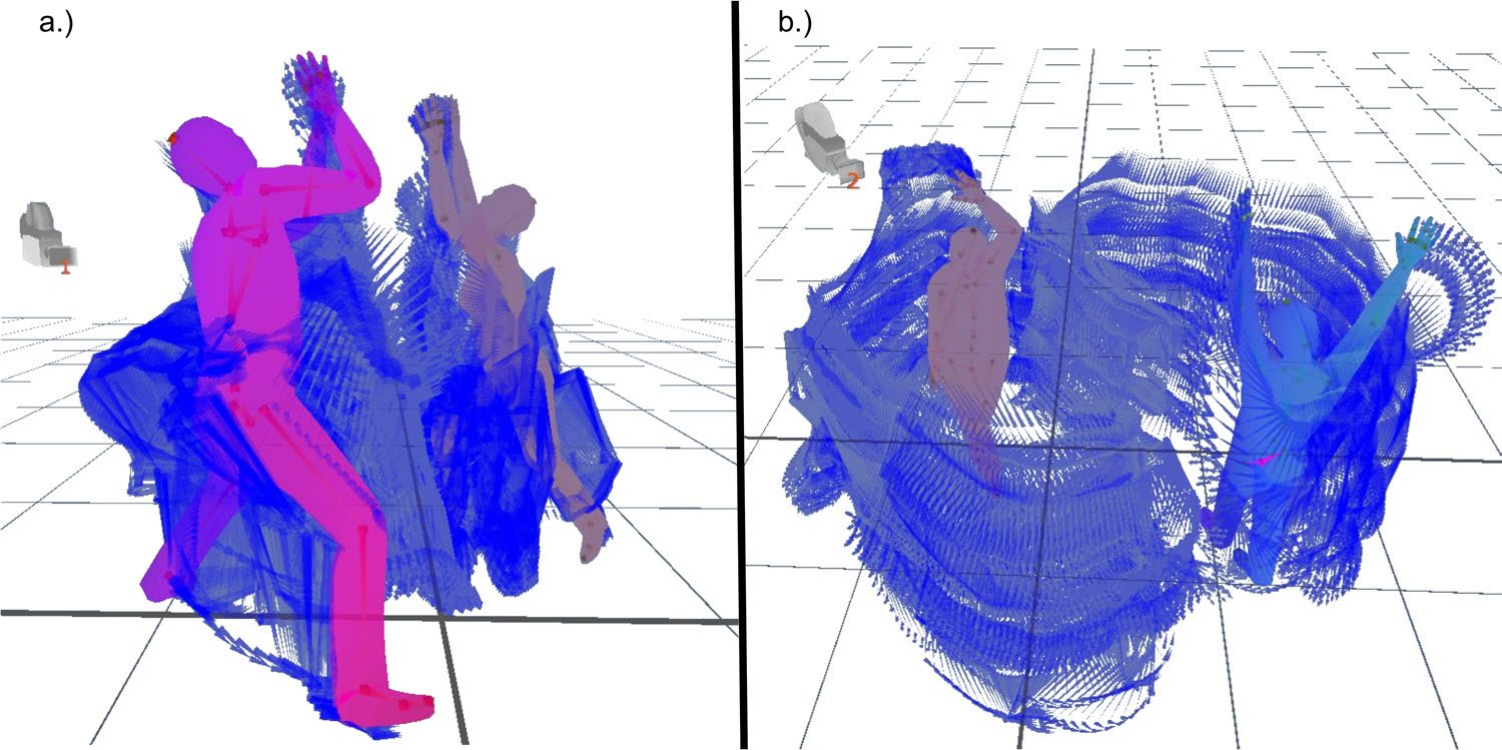
Two examples of behavioural imaging data during the BCT. A single frame of data is shown in the full outline of the individuals in a dyad, shown with 10 seconds of previous data (300 frames). (**a**) An imaging sample from dyad #32. (**b**) An imaging sample from dyad #29.

We designed an experimental task that specifically allows the measurement of self-organization dynamics in spontaneous individual and interactive movement: the *Body-Conversation Task (BCT)*. The BCT consists in a 5-minutes dyadic movement improvisation task, in which two participants are asked to “have a conversation without words”: participants are thus asked to interact and communicate by improvising movement with their whole body (see Fig. 1 for illustration of behavioural imaging data during the BCT; see also Supplementary Video S1 for a 30-second sample of these data). The development of this experimental task is embedded in and arises from a more general novel research approach in movement research, which aims at studying movement dynamics in its full complexity (see for example^38,39,51–53^). More specifically, the BCT draws on three important methodological advances compared to previous research on interpersonal synchrony. First, the common use of minimal settings - e.g. periodic movements of only hands or fingers^52,54,55^ might not do justice to the complexity of movement dynamics as they occur in natural human interactions. The BCT, in contrast, allows for the observation of spontaneous whole body movement. Second, interpersonal synchrony has often been studied in the context of verbal conversations^38,40,56,57^. Part of the interactive negotiation thus involves both spoken language and movement, which makes it difficult to differentiate their respective contribution to it. By excluding the possibility of spoken language, the BCT ensures that the interactive dynamics are entirely expressed in one modality only, i.e. through movement. Third, movement dynamics are often observed within pre-defined contexts, such as collaborative^32^ and mirroring tasks^51,52^ or friendly or argumentative conversation^40,57^. In such contexts individuals’ behaviour and movement are still mostly constrained by pre-determined goals or rules^56^. In contrast, BCT provides a social setting with minimal social constraints, and thus allows for the emergence of spontaneous and self-initiated behaviour.

Methodological advances such as a focus on whole-body movement, absence of spoken language and minimal social constraints have been partially (or independently) put forward in experimental tasks of previous research. However, these three aspects have not yet been combined, apart from one recent notable exception^53^. With the BCT we propose that bringing together all three of these aspects allows to validly capture the complex dynamics of self-organized behaviour, both at the social as well as individual level.

**Figure 2:**
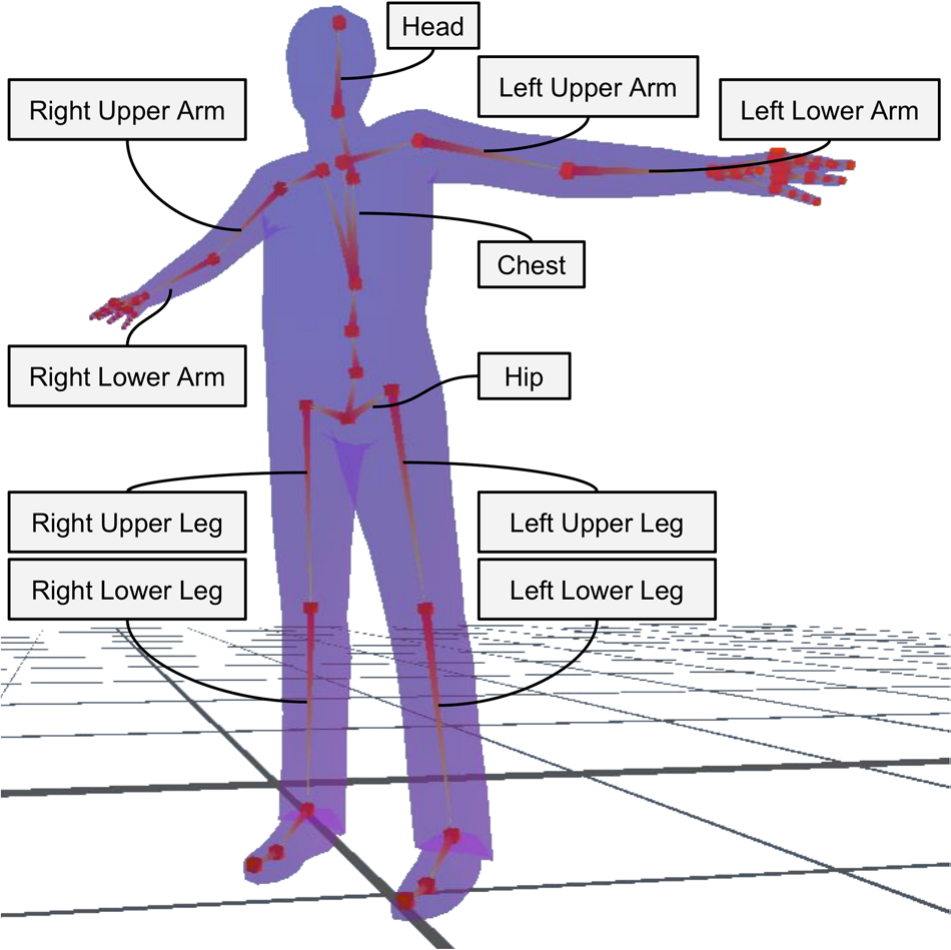
The 11 limb segments tracked in behavioural imaging. This figure also illustrates the T-Pose assumed by participants at the beginning of each recording.

In order to account for the dynamics of individual and interactive movement, we applied behavioural imaging technology (see Fig. 2 for limb segments tracked with behavioural imaging). We simultaneously measured spontaneous, whole-body movement of two individuals as multivariate systems. Thereby we were able to characterize both individual and dyadic self-organization with two parallel measures, i.e., intrapersonal and interpersonal movement synchrony. These measures utilized an average of time-lagged windowed cross correlations of all possible pairwise combinations of limb segments within the individual or between individuals in a dyad (see Supplementary Video S2 for a sample of behavioural imaging data time-lagged and windowed).

Based on these methodological and technological advances, in this study we widened the focus of research on interpersonal synchrony and addressed the multilevel interrelation between social and individual forms of self-organization. We specifically focused on psychological forms of individual self-organization and asked: how do social dynamics of interpersonal synchrony affect psychological processes of self-regulation? Importantly however, in addressing this question we also took into account behavioural processes of individual self-organization, measured as movement synchrony among the individual’s limb segments.

66 previously unacquainted adults took part in the study in pairs. Both participants separately went through a standardized movement warm-up before the BCT (see Supplementary Information for full instructions). Immediately after the warm-up, they took part in the BCT and were tracked with behavioural imaging technology (see Method). Before and after the movement part of the experiment (warm-up and BCT) each participant separately filled in brief self-report questionnaires assessing positive affect and self-regulation of affect (see Method for details on measures).

We first confirmed the presence of interpersonal and intrapersonal synchrony and we examined the relationship between interpersonal synchrony and positive affect in replication of previous research. These steps aimed to validate the BCT in facilitating interactive phenomena similar to previous experimental designs. We then turned to our main research question and investigated the effect of interpersonal synchrony on the self-regulation of affect. We drew on the enactive hypothesis^10^ that the human self, for its stability and regulation, needs to balance social attunement with disengagement. We thus hypothesized that interpersonal synchrony’s known tendency to increase positive affect would have a trade-off: higher synchrony with others would predict more difficulties in the self-regulation of affect (the trade-off hypothesis). Although our hypothesis aimed to test the impact of dyad-level synchrony on the self, we also accounted simultaneously for intrapersonal synchrony: we investigated the impact of intrapersonal synchrony as well as of the relationship between inter- and intrapersonal synchrony both on positive affect and on the self-regulation of affect.

## Results

### Movement dynamics during the BCT

We described the characteristics of the calculated movement dynamics. Regarding synchrony values, intrapersonal synchrony, *M*(SD) = 0.12 (0.025), was larger than interpersonal synchrony in the sample, *M*(SD) = 0.09 (0.018), *t*(65) = 12.61, *p* < 0.001, *d* = 1.55.

We then explored the correlations among movement variables at the individual and dyad levels (Table 1). Raw amount of movement across the task (average velocity) and variability of movement (average standard deviation of limb segments over time) were assessed for each individual and averaged for dyads. Table 1 provides these bivariate correlations. Synchrony values were largely unrelated to the other movement measures, as there was only a small negative correlation between an individual’s level of intrapersonal synchrony and their average velocity of movement, *r*(64) = −0.25, *p* = 0.045. This means that those who moved more tended to demonstrate less intrapersonal synchrony. There were significant correlations between the self’s and the other’s average velocity of movement, *r*(31) = 0.63, *p* = 1.38 * 10^−8^, and between the self’s and the other’s average variability of movement across limb segments, *r*(64) = 0.38, *p* = 0.002. Similarly, one’s individual intrapersonal synchrony was highly correlated with the other’s intrapersonal synchrony in the interaction, *r*(31) = 0.71, *p* = 3.51 * 10^−11^. Paired participants thus adapted to each other not only in terms of how much or how diversely they moved, but also in terms of the coherence of their movement within themselves (intrapersonal synchrony).

**Table 1:** Relationships among individual and dyad-level synchrony and movement variables with confidence intervals.

Variable	1	2	3	4	5	6	7	8
1. Intrapersonal synchrony								
2. Interpersonal synchrony (Dyad)	0.83**							
	[0.74, 0.90]							
3. Other’s intrapersonal synchrony	0.71**	0.83**						
	[0.56, 0.81]	[0.74, 0.90]						
4. Velocity	−0.25*	−0.09	−0.09					
	[−0.46, −0.01]	[−0.32, 0.16]	[−0.32, 0.16]					
5. Dyad’s velocity	−0.18	−0.10	−0.18	0.90**				
	[−0.41, 0.06]	[−0.43, 0.25]	[−0.41, 0.06]	[0.85, 0.94]				
6. Other’s velocity	−0.09	−0.09	−0.25*	0.63**	0.90**			
	[−0.32, 0.16]	[−0.32, 0.16]	[−0.46, −0.01]	[0.46, 0.76]	[0.85, 0.94]			
7. SD	−0.16	−0.10	−0.01	0.80**	0.67**	0.41**		
	[−0.39, 0.08]	[−0.33, 0.15]	[−0.25, 0.24]	[0.68, 0.87]	[0.51, 0.78]	[0.19, 0.60]		
8. Dyad’s SD	−0.10	−0.12	−0.10	0.73**	0.81**	0.73**	0.83**	
	[−0.33, 0.15]	[−0.44, 0.24]	[−0.33, 0.15]	[0.59, 0.82]	[0.64, 0.90]	[0.59, 0.82]	[0.74, 0.89]	
9. Other’s SD	−0.01	−0.10	−0.16	0.41**	0.67**	0.80**	0.38**	0.83**
	[−0.25, 0.24]	[−0.33, 0.15]	[−0.39, 0.08]	[0.19, 0.60]	[0.51, 0.78]	[0.68, 0.87]	[0.15, 0.57]	[0.74, 0.89]

All correlations were calculated with the Pearson method. Where correlations involve two dyad-level variables, *df* = 31; for all other pairs, *df* = 64. “Velocity” indicates average total velocity per frame. “SD” indicates average of the limb segment-wise standard deviations of velocity. Values in square brackets indicate the 95% confidence interval for each correlation. The confidence interval is a plausible range of population correlations that could have caused the sample correlation^69^. *Indicates *p* < 0.05. **Indicates *p* < 0.01.

### Validating the BCT: synchrony was present and predicted increases in positive affect

We first sought to validate the ability of BCT to facilitate spontaneous individual and interactive movement by assessing for the presence of intrapersonal and interpersonal synchrony over limb segment level data randomly shuffled within 10-second windows. We then conducted paired t-tests examining the mean difference of synchrony values between the original and the randomly shuffled data. There was a very large effect of intrapersonal synchrony over shuffled data, *t*(65) = 23.43, *p* = 1.89 * 10^−33^, *d* = 2.89, and likewise for interpersonal synchrony, *t*(32) = 15.52, *p* = 1.91 * 10^−16^, *d* = 2.70.

We then examined shifts in positive affect following the BCT and their relationship to synchrony dynamics for further validation of the task on participant experience. Positive affect was measured with the Positive and Negative Affect Scale (PANAS, German version), a brief self-report questionnaire assessing momentary affect^58^. There was a significant increase in positive affect following the BCT, *t*(65) = 5.32, *p* = 1.39 * 10^−6^, *d* = 0.65. Using linear mixed effects modelling, we built a series of models predicting positive affect after the BCT with dyads as a random effect. We controlled for pre-task affect at the first step, then added the individual-level effect of intrapersonal synchrony at a second step, followed by the dyad-level effect of interpersonal synchrony at a third step (full main effects model). All variables were centred and standardized for all analyses.

We found significant effects of both inter- and intrapersonal synchrony, showing divergent relationships with positive affect. In the full main effects model, we found that intrapersonal synchrony predicted a reduction in positive affect, *B* = −0.45, *SE* = 0.14, *t* = −3.16, *p* = 0.0016, while interpersonal synchrony predicted increased positive affect, *B* = 0.31, *SE* = 0.14, *t* = 2.17, *p* = 0.03. The positive effect of interpersonal synchrony confirmed findings in previous research, thus validating the BCT. The opposing effect of intrapersonal synchrony (included for completeness of the model) was, however, novel and unexpected. There were slight, but statistically nonsignificant effects of interpersonal synchrony associated to reductions in negative affect, *B* = −0.22, *SE* = 0.17, *t* = −1.34, *p* = 0.18. As with the positive affect analysis, this was considered over and above the effect of intrapersonal synchrony, which did not have a statistically significant impact on negative affect, *B* = 0.10, *SE* = 0.15, *t* = 0.67, *p* = 0.50.

### The trade-off hypothesis: interpersonal synchrony predicted a reduction in self-regulation of affect

Since interpersonal synchrony generated within the BCT appeared to reliably predict an increase in positive affect, we turned to testing the trade-off hypothesis, which states that interpersonal synchrony would also predict a reduction in the individual’s ability to self-regulate affect.

#### Synchrony and general self-regulation

Self-regulation of affect is a specific case of self-regulation, so we first tested the link between synchrony and general self-regulation as a useful context to our hypothesis. We broadly assessed self-regulation across two general measures. The State Difficulties in Emotion Regulation Scale (S-DERS)^59^ was used to measure general difficulties in emotion regulation and the State Self-Control Capacity Scale (SSCCS; German adaptation) was used as an indicator of self-control strength^60^. For all self-regulation measures, we followed the same hierarchical model strategy as above, stepwise building up to the central test of the effect of interpersonal synchrony on self-regulation in the context of dyadic variation, pre-task self-regulation, and intrapersonal synchrony.

Neither intrapersonal synchrony nor interpersonal synchrony predicted general post-task emotion regulation difficulties scores (S-DERS Scale) in hierarchical models (ps > 0.4). We then tested the impact on self-control strength (SSCCS Scale) and we used the same pattern of models to examine their relationships with intra- and interpersonal synchrony. We found no independent effect of intrapersonal synchrony on post-task self-control (p = 0.19), and detected some small negative relationship with interpersonal synchrony, B = −0.27, SE = 0.16, t = −1.71, p = 0.087.

#### Synchrony and self-regulation of affect

We conceived of self-regulation of affect as a dimension of self-regulation occurring at the pre-reflective and immediate level. We thus assessed self-regulation of affect with the modulate subscale of the State Difficulties in Emotion Regulation Scale (S-DERS), which targets felt difficulties in the implicit modulation of affect^51^. Setting up the steps of the model for the main test of our hypothesis, we entered pre-task modulate scores and the random effect of dyads at the first step in predicting post-task modulate scores. At the second step, we added individual-level intrapersonal synchrony, which did not significantly predict post-task modulate scores, *B* = 0.01, *SE* = 0.06, *t* = 0.10, *p* = 0.93.

**Figure 3:**
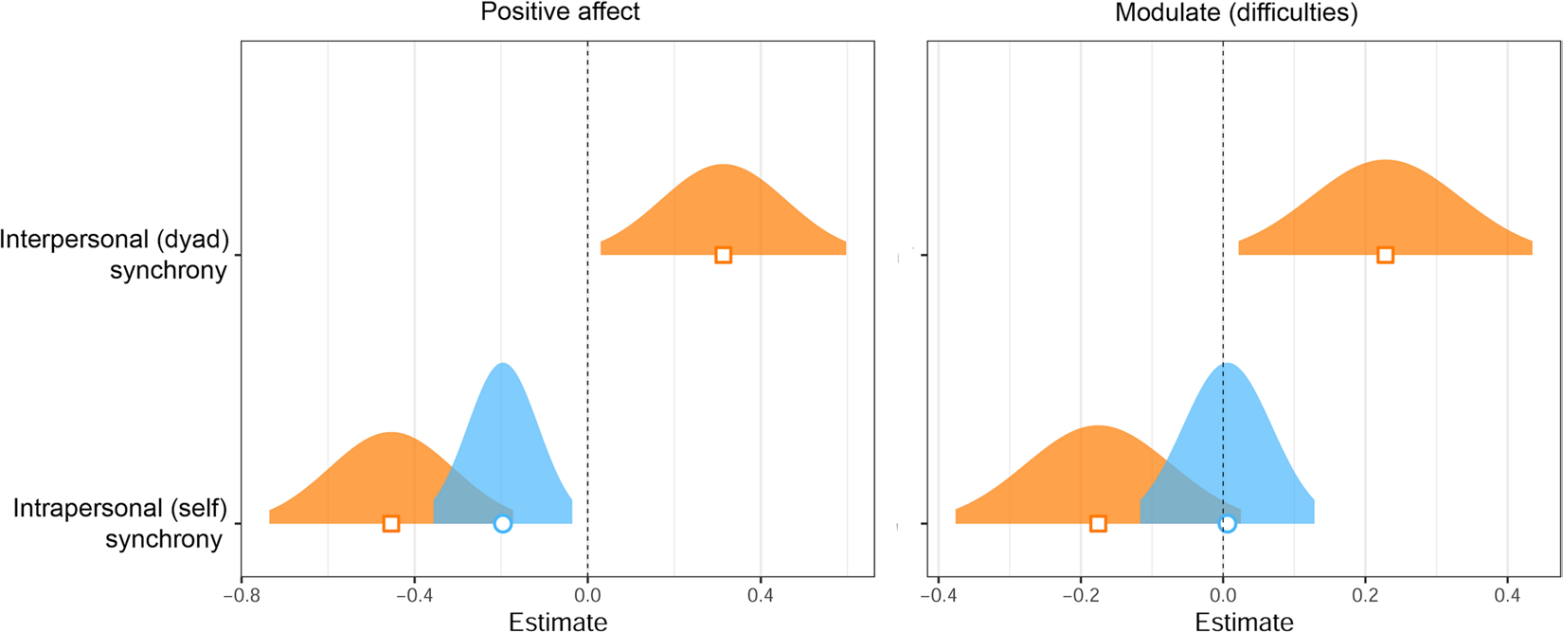
Fixed effects of synchrony on post-BCT positive affect and difficulties with modulation of affect. These depict synchrony fixed effects of two multilevel linear mixed effects models. Not pictured are control variables of each model: pre-BCT values of respective dependent variable and the random effect of dyad assignment. *Positive affect* refers to the PA subscale of Positive and Negative Affect Scale and *Modulate (difficulties)* refers to the Modulate subscale of State-Difficulties in Emotion Regulation Scale, scored so that higher values indicate more difficulties with self-regulation of affect. Blue (circle) indicates effects at the individual-level intrapersonal synchrony step. Orange (square) indicates effects at the dyad-level interpersonal synchrony step (full main effects model). 95% confidence distribution is provided for each effect.

**Figure 4:**
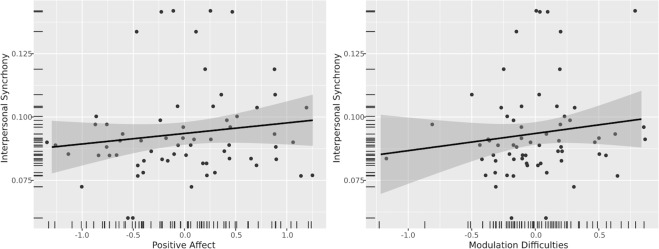
Relationships of interpersonal synchrony with positive affect and modulation difficulties accounting pre-BCT scores on respective measures, random effects of dyad, and the effect of intrapersonal synchrony.

At the third step, in a crucial test of the trade-off hypothesis, we entered dyad-level interpersonal synchrony into the model. Interpersonal synchrony significantly predicted difficulties in modulation of affect after the BCT, *B* = 0.23, *SE* = 0.12, *t* = 2.17, *p* = 0.03. We found support for the hypothesis that positive affect trades off with self-regulation of affect during interpersonal synchrony. See Fig. 3 for forest plots of the modelled effects of interpersonal and intrapersonal synchrony on positive affect and affect modulation. Also see Fig. 4 for scatter plots of interpersonal synchrony on positive affect and affect modulation accounting for all other variables in their respective models.

Surprisingly, intrapersonal synchrony became marginally significant in the opposite direction at this third step, *B* = −0.18, *SE* = 0.10, *t* = −1.72, *p* = 0.086. Intrapersonal synchrony tended to predict fewer difficulties in affect modulation when considering the effect of interpersonal synchrony. However, interpersonal synchrony still conveyed a stronger effect on affect modulation (Fig. 3). In a fourth step, we explored the interaction effect between interpersonal and intrapersonal synchrony in predicting post-task affect modulation. There was no interaction effect, *p* = 0.68. Accounting for the interaction effect did not change the independent effects of the variables (see Table 2 for a full report of this model).

**Table 2:** Full linear mixed effects model testing trade-off hypothesis of difficulties in self-regulation in affect.

*Predictors*	*Estimates*	Modulate (difficulties) Post-BCT											
		*CI*	*p*	*Estimates*	*CI*	*p*	*Estimates*	*CI*	*p*	*Partial R* ^2^	*Estimates*	*CI*	*p*
Intercept	0	−0.12–0.12	1	0	−0.12–0.12	1	0	−0.12–0.12	1		0.02	−0.13–0.16	0.829
Modulate (difficulties) Pre-BCT	0.9	0.80–1.01	<0.001	0.91	0.79–1.02	<0.001	0.89	0.78–1.00	<0.001	0.789	0.89	0.78–1.00	<0.001
Intrapersonal synchrony				0.01	−0.12–0.13	0.923	−0.18	−0.38–0.02	0.086	0.073	−0.17	−0.38–0.03	0.092
Interpersonal synchrony							0.23	0.02–0.43	**0.03**	0.185	0.25	0.02–0.47	**0.03**
Interaction of Intra- and interpersonal synchrony											−0.02	−0.11–0.07	0.681
*Random Effects*													
σ^2^	0.17			0.17			0.15				0.15		
τ_00_	0.05_Dyad_			0.05 _Dyad_			0.05 _Dyad_				0.06 _Dyade_		
ICC	0.22 _Dyad_			0.22 _Dyad_			0.26 _Dyad_				0.28 _Dyad_		
Observations	66			66			66				66		
Marginal R^2^/Conditional R^2^	0.794/0.839			0.792/0.837			0.802/0.854				0.800 / 0.855		

R^2^ were computed using standardized generalized variance approach^70^. Test of trade-off hypothesis in bold. 95% CI reported.

We sought to rule out alternative influences of movement qualities and demographic variables on self-regulation of affect. These movement qualities involved individual and dyad-level calculations of raw amount of movement (average velocity) and variability of movement (average standard deviation of limb segments over time). Due to high multicollinearity between dyad-level and individual-level velocity (VIF > 10, *r* = 0.90), we examined the impact of velocity and variability as additional fixed effects in two separate models: an individual-level model and a dyad-level model. We found that neither model substantially altered the significance nor effect size of interpersonal synchrony on affect modulation difficulties (dyad-level: *B* = −0.20, *SE* = 0.11, *t* = 1.82, *p* = 0.03; individual-level: *B* = −0.19, *SE* = 0.11, *t* = 1.67, *p* = 0.03) and that none of the new variables were significant predictors (*p*s > 0.5). Neither model improved prediction over the original model, *p*s > 0.7.

We also explored the role of gender, gender composition of the dyad (same or different), nationality, and prior experience with a movement practice on interpersonal and intrapersonal synchrony in a series of t-tests. Only gender composition of the dyad demonstrated differences in any synchrony, and only for interpersonal synchrony, *t*(64) = 2.43, *p* = 0.02, *d* = 0.60. Different gender dyads had more interpersonal synchrony, *M(SD)* = 0.10 (0.02), than same gender dyads, *M(SD)* = 0.09 (0.01). Adding gender composition to the trade-off hypothesis model did not have any impact on the relationship of interpersonal synchrony with self-regulation of affect, did not itself predict self-regulation of affect, nor interact with interpersonal synchrony to predict self-regulation of affect. Such was the case for all of the demographic variables in predicting both self-regulation of affect and positive affect.

## Discussion

We investigated for the first time the effect of social self-organizing dynamics, measured as interpersonal movement synchrony, on the self-regulation of affect. Self-regulation of affect was measured as an index of psychological processes of self-organization at the individual level. A measure of positive affect was also used to validate our experimental design in replication of previous research. We drew on a recently formulated hypothesis in enactive cognitive science, which suggests that too much social attunement might be detrimental for the self, and consequently formulated the so-called trade-off hypothesis. We expected to confirm previous evidence that interpersonal synchrony increases positive affect; yet we predicted that this comes with a trade-off for the self, that is, interpersonal synchrony would also predict a decrease in a person’s ability to self-regulate affect. Interpersonal movement synchrony was measured during a novel dyadic movement task (Body-Conversation Task; BCT), devised to facilitate spontaneous social interactions. Using multivariate behavioural imaging methodology, we were also able to fully account for individual-level intrapersonal synchrony occurring concurrent to dyad-level interpersonal synchrony. Although our main hypothesis tested the impact of dyad-level self-organization on the psychological processes of self-regulation, the measurement of intrapersonal synchrony allowed for the additional consideration of behavioural processes of individual-level self-organization in our models. The missing of a control condition in our design impeded the establishment of causal relationships. Yet, the standardized design, statistical control and high-resolution data allowed making accurate predictions on the relation between behavioural processes and psychological variables.

We first validated that the BCT facilitated both intrapersonal and interpersonal synchrony. Consistent with previous findings, we also found that participants who synchronized more with their partner showed a significant increase in positive affect after the BCT. We then proceeded to test the hypothesis that this increase in positive affect occurred at a trade-off for the self. Higher interpersonal synchrony during the BCT predicted greater difficulties in self-regulation of affect, measured as implicit modulation of affect (S-DERS modulate subscale). These results supported the trade-off hypothesis given that the processes influencing both positive affect and affect regulation occurred concurrently (i.e., during the period of the BCT). The same test on more general measures of self-regulation did not yield significant results. We did not find evidence that interpersonal synchrony impacted general emotion regulation (S-DERS total score). This suggested some specificity to the effect of interpersonal synchrony on affect modulation rather than of other processes involved in self-regulation, such as for instance self-acceptance and self-awareness. Such reflective processes are less likely to be influenced by implicit, bodily dynamics than the pre-reflective processes captured by the S-DERS modulate subscale. The measure of momentary self-control (SSCCS scale) showed a pattern of results similar to our main hypothesis test: a diminished capacity for self-control was predicted by higher interpersonal synchrony although with a weaker, non-significant effect. The analysis of intrapersonal synchrony showed an interesting and novel result. We found a tendency for intrapersonal synchrony to oppose the effects of interpersonal synchrony on positive affect and self-regulation of affect: higher intrapersonal synchrony predicted diminished positive affect and (albeit only marginally) improved modulation of affect. These findings also point to the potential divergent effect that such movement dynamics have respectively on affect and self-regulation of affect.

First of all, the results of our study provide new evidence for the strong link between social self-organizing dynamics and individual self-organizing dynamics. We found that interpersonal synchrony has a stronger relationship with the self-regulation of affect than the individual’s intrapersonal synchrony during the BCT. A possible explanation for this could be that the self-organizing force of the interaction overrides the self-organizing force of processes at the bodily, individual level. Thus, and perhaps surprisingly, the human capacity for self-regulation might depend more on social interaction dynamics than on individual processes. It has been widely recognized that interpersonal engagements and synchrony are vital for the regulation of the self in the early developmental stages^61,62^. However, until now, the role of interpersonal synchrony for the regulation of the self past this initial phase has remained unclear. Our study demonstrates that interpersonal synchrony may have a strong impact on a person’s self-regulation even beyond infancy, i.e. adults too, strongly rely on social interactions for self-regulating and modulating their own affect.

Our findings thus speak to the deeply social nature of the human self^10,63^. But they also show more precisely that the strong entrainment of the self in social dynamics can have a negative impact when it comes to a person’s self-regulatory abilities. When we rely on others to regulate our emotions, we outsource some of the load of our self-regulatory effort onto the interaction^64,65^. This tendency to socially outsource self-regulatory processes might explain the link between interpersonal synchrony and self-regulation: the more we rely on the interaction the less we may feel in control and able to self-regulate on our own. Harmony with others therefore has a trade-off: while it feels good to be in sync, we also pay a price for this, in that we are less efficient when it comes to regulating our own emotions. Interestingly, we found preliminary evidence for a similar trade-off of positive affect and self-regulation with intrapersonal synchrony: more synchrony within a person’s body tended to be associated with a better capacity to self-regulate emotions and yet it also tended to temper positive affect. Further research is needed to explore these dynamics as well as the dynamics that may exist between interpersonal and intrapersonal synchrony in how they mutually impact self-regulation over time.

Support for the trade-off hypothesis shows that the impact of synchrony on the self is not straightforwardly good or bad. Instead it suggests a more dynamical outlook and nuanced understanding of the relation between the interactive and individual level. Our findings provide new evidence for the enactive hypothesis that self-organizing processes at both the individual and the social level play a crucial role for the stability of the self. Indeed, the human self seems to require both independence from as well as entrainment in social relationships^10^. The enactive approach emphasizes that a lack of either of these aspects can be detrimental to the self. There is much evidence supporting the claim that a lack of social engagement has a negative impact on our well-being^48,49,66^. The novelty of our outcomes consists in showing that too much social attunement, however, may also become a risk for the self. Openness to and harmony with others is not all we need for stability. We need to balance our reliance on others with separation and independence from them.

Our results point to the need of further investigating and better understanding the relation between self-organizing social dynamics and self-regulatory processes within the self, particularly at the pre-reflective level. The methodological and technological advances applied in this study constitute an important step towards this goal. Specifically, future work should explore the possibility of simplifying our methodology and data processing to make this approach more widely accessible to basic and applied contexts. Basic research on the impact of social interaction dynamics on the self-regulatory processes of the self has also relevant applied and societal implications such as psychotherapy process and conflict resolution.

Finally, our findings have crucial implications for the way we conceive of the human self and its relation to the social world. Humans are indeed no islands: the self is a thoroughly social entity. Yet, it is not as simple as that. In this study we have provided evidence that even at the basic level of spontaneous movement interactions, we also need disengagement and separation from others in order to self-regulate and maintain our stability.

## Method

### Participants

Participants were recruited through an announcement on the participants portal of the Technical University of Berlin (TUB). Inclusion criteria were: age between 20 and 35, capacity for light movement of the whole body and knowledge of the German and English language. Participants were randomly paired as they signed up for the study. Upon disclosure of the partner’s name, all participants confirmed that they had never met their partner nor heard their name.

72 participants took part in the study in exchange for monetary compensation. After three pilot dyads, the Body-Conversation Task (BCT) instructions were revised. These 3 dyads were excluded from the data analyses presented in this report. A total of 66 participants were therefore included in the study (45 female, 21 male). 18 (54.55%) dyads were of same gender and 15 (45.45%) of opposite gender. The mean age was 27.5 ± 4.0 years. 55 participants were of German nationality, 5 of other European nationalities and 6 of Non-European nationalities. Only 42 (63%) participants were university students. There were no professional dancers in the sample. 36 (54.54%) participants declared that they did not participate in a regular sport or dance activity or did so only sporadically (less than once per week), 30 (45.45%) did regularly or intensively practice sport or dance (every week). The study was approved by the Ethics committee of the TUB and conducted in accordance with the Declaration of Helsinki. All participants gave written informed consent before taking part in the study.

### Setup and procedure

**Figure 5:**
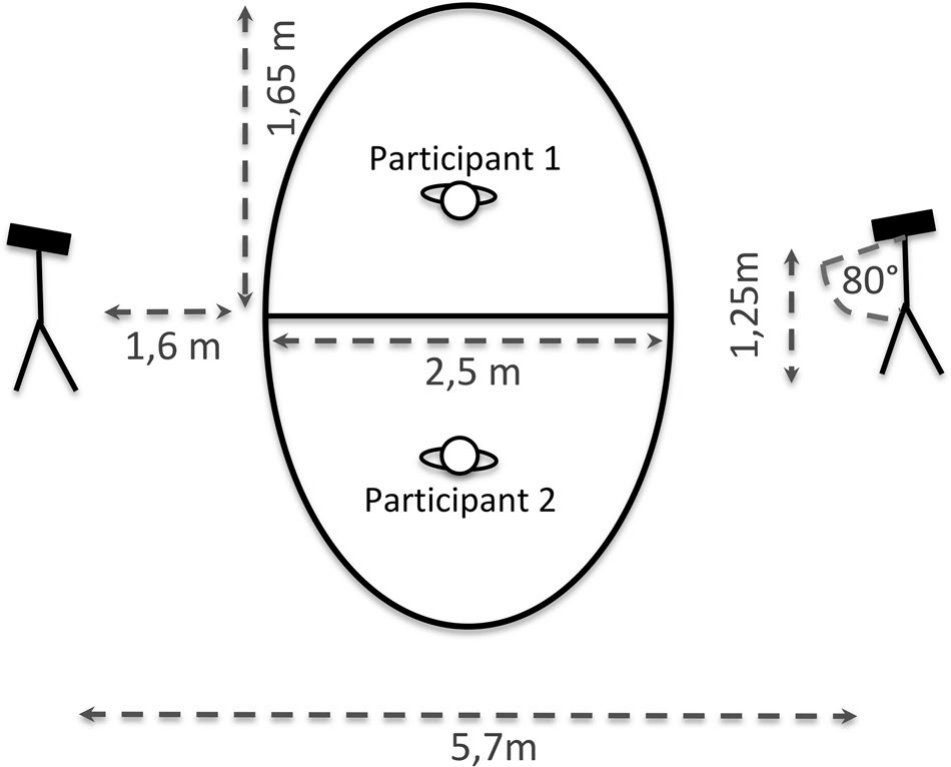
Setting of the Body-Conversation Task (BCT). Each participant could move in a semi-elliptical space of 2.5 m width and 1.65 m depth. Cameras were placed at a 1.6 m distance from the ellipsis and at a 5.7 m distance from each other. They were oriented at 80° to the vertical and at 1.25 m height from the ground.

The experimental procedure took place in one room in which paired participants met for the first time on the day of the experiment. The room’s layout is depicted in Fig. 5. Two Kinect version 2 cameras were placed facing each other at the two sides of the room at a distance of 5.7 m. An elliptical form 2.5 m × 3.3 m was drawn on the floor and divided in two half-ellipses. It designated the area in which the bodily warm-up and the BCT took place (Fig. 1 for a depiction of movement). Light conditions were kept stable at all times by darkening the windows and using the same artificial light only.

Upon arriving, participants read and signed the informed consent form. Participants were then invited to sit separately and complete brief state questionnaires about their momentary affect and self-regulation of affect. They were then invited to step into the ellipsis drawn between the two cameras and marking the reliable capture area of the behavioural imaging system (Fig. 5). There, they first took part in the preparatory bodily warm-up. For the warm-up, participants stood, in their respective half of the ellipse facing each other backwards, looking at a wall. For 5 minutes, the experimenter guided them through simple warm-up exercises by reading instructions from a script (see Supplementary Information). The movement warm-up consisted in simple and progressive movements of all body parts and in the active exploration of the person’s movement possibilities. Because participants faced the wall and were not allowed to turn and look at the other participant during the entire warm-up, we were able to avoid a bias towards unintended synchronization of movement prior to the BCT. For the behavioural imaging system, at the beginning of each recording, participants needed to assume a T-pose, thus standing upright with their legs together and their arms parallel to the floor like the letter “T” (Fig. 2). The warm-up sessions were recorded, but the obtained imagining data was not used in the current study.

Next, the experimenter read to participants the instructions for the BCT (see Supplementary Information). Participants were now asked to improvise movement together and to communicate with the other person without using words. They were encouraged to do so by moving the whole body (not only pantomime with hands) and by moving at all times (thus diminishing the effect of conversational turn-taking). Participants were allowed to move freely within their space (one half-ellipsis) but not to step out of it, nor to step into the space of the other person. In order to optimize imaging accuracy, participants were instructed to remain on their feet and also to refrain from touching the other person. The experimenter answered any questions that participants had about the task. When answering, the experimenter clarified what was already entailed in the instructions without giving further information. Participants were also informed that the experimenter would stay in the room but turn her chair to the wall and therefore not observe their interaction. Finally, the experimenter started the recording with the following final instruction: “Improvise movement together with the other person. Imagine you are having a conversation without words, only through movement”. She gave the start for the interaction and after 5 minutes she stopped the interaction. Immediately upon completion of the task, participants were invited to once again, separately complete the brief state questionnaires on momentary affect and self-regulation of affect. The experiment lasted approximately 50 minutes. After these initial 50 minutes, the experiment continued with an additional part investigating participants’ experience (not included in the current study). The instructions for the BCT were changed after the pilot phase (3 experiments). Initially participants were instructed to “try to get to know the other person through movement”. As a consequence, research participants mainly used hands movements and pantomime to communicate specific personal information. Since the BCT was supposed to facilitate whole body expressive movement we thus changed the instructions into the more open format of “improvising movement together.”

### Subjective measures

Participants completed the following questionnaires for assessing their psychological state immediately before and immediately after the movement part of the experiment.

#### Positive affect

We measured positive affect with the German version of the Positive And Negative Affect Scale (PANAS)^58^. The PANAS consists of 20 adjectives that describe different emotions: 10 adjectives describe positive affect and the other 10, negative affect. Participants rated each adjective on a 5-point Likert scale according to the degree of correspondence to their current emotional state. Given its importance in previous research on interpersonal synchrony, for this study we focused on positive affect (PA subscale), though we also included an analysis of the NA scale.

#### Self-Regulation of affect

To assess self-regulation of affect, which was central to our trade-off hypothesis, we used the modulate subscale of the State Difficulties in Emotion Regulation Scale (S-DERS)^59^. The S-DERS is a self-report measure of momentary emotion dysregulation based on 21 items rated on a 5-point Likert scale. Higher scores correspond to more difficulties in emotion regulation. It includes 4 subscales: non-acceptance, awareness, clarity and modulate. The modulate subscale assesses the pre-reflective capacity to modulating emotional and behavioural responses at a particular moment. The subscale’s items thematically capture the feeling of being overwhelmed versus the feeling of being in control. In contrast, the other 3 subscales of the S-DERS instrument refer to the person’s reflective stance towards her own emotions, i.e. how aware she is and how much clarity and acceptance she has about her current emotional state. For the test of the trade-off hypothesis we focussed on the modulate subscale and thus defined self-regulation of affect as the modulation of emotions at the immediate and pre-reflective level in the moment. Since the modulate subscale captures self-regulation processes at the pre-reflective level, we expected that it would be more sensitive to the impact of synchrony dynamics.

An assessment of general self-regulation, mainly targeting aspects such as the ability of self-reflection and self-control, was included as context to the main hypothesis. The S-DERS total score and the German adaptation of the State Self-Control Capacity Scale (SSCCS)^60^ were used to measure general self regulation. The SSCCS is a measure of momentary available self-control strength. It is a one-dimensional scale, which includes 25 items rated on 7-points – the higher the total score, the higher the capacity for self-control. The SSCCS scale has shown high reliability (α = 0.93). Both the S-DERS total score and the modulate subscale have also demonstrated good internal consistency (respectively, α = 0.86 and α = 0.85).

### Behavioural imaging

We utilized an active motion capture system that does not require attaching any sensors or markers on the participants’ bodies. The two Kinect cameras recorded concurrent depth data of both participants. Using iPiSoft Motion Capture Studio, these depth data recordings were merged, oriented in three-dimensional space in a global reference frame, and used as raw data from which the movement of these two individuals were tracked. In contrast to previous research with Kinect cameras, which directly records from the Kinect to the hard drive, our approach synthesized the depth camera data from both cameras (~6 GB of depth information over time). At a separate step, these synthesized videos were intensively modeled and revised in the iPiSoft Studio software using automatic algorithms. Supplementary Video S1 exemplifies the output of this modelling process, depicting the data from which all of our movement calculations derive. In pretesting, we found our approach to be dramatically more reliable than directly recording Kinect location data for modelling free, spontaneous movement of the body as a whole system. Movement was tracked on 11 limb segments at 30 Hz for each person as radians per second. Imaged limb segments included the following: head, chest, hip, upper arms (x2), lower arms (x2), upper legs (x2), and lower legs (x2) (see Fig. 2 for a depiction). Biomechanical data were then imported into R, converted into magnitude radians per second, and filtered using optimal parameters derived against a gold standard passive motion capture system (a second order low-pass Butterworth filter at a digital filter value of 0.24; see^67^, p. 46). Observations were removed and spline interpolated if they exceeded 20 times the interquartile range for a given limb segment of a participant. These instances were extremely rare (0.0049% of observations).

### Calculation of synchrony

Intrapersonal and interpersonal synchrony were both calculated as windowed cross-correlations^26,68^ among unique combinations of limb segment pairs. All 900 cross-correlations in a window of 900 observations at +/− 5 seconds lags were used (see Supplementary Video S2 for an illustration of the interaction with 10 second of movement data displayed). The absolute values of these cross-correlations were transformed to Fisher’s Z and aggregated within intervals of 30 seconds and then across all intervals of the experimental procedure duration. For intrapersonal synchrony, all within-person limb segment pair combinations (55 pairs) were calculated. For interpersonal synchrony, all between-person segment pair combinations were calculated (121 pairs). Thus, the extent of all possible unique limb segment pairs was accounted for, both within-person (intrapersonal synchrony) and between persons (interpersonal synchrony) without any overlap in the calculations between these two measures. We assessed for the presence of synchrony by randomly shuffling limb segment velocity data in 10 second windows. We then performed the same analyses of intrapersonal and interpersonal synchrony, but correlating original with shuffled data.

## Supplementary information

Supplementary Information

Supplementary Video S1

Supplementary Video S2

## Data Availability

The datasets generated and analysed during the current study are available from the corresponding authors on reasonable request.
