# Supplementary material for: Interpersonal synchrony feels good but impedes self-regulation of affect
Source: Sci Rep. 2019 Oct 11;9:14691. doi: 10.1038/s41598-019-50960-0 (PMC6789117; doi:10.1038/s41598-019-50960-0)
Supplement: Supplementary file 1 — Supplementary Information [file 41598_2019_50960_MOESM1_ESM.pdf]

Interpersonal synchrony feels good but impedes self-regulation of affect

Laura Galbusera, Michael T. M. Finn, Wolfgang Tschacher and Miriam Kyselo

Supplementary Information

Supplementary Material S1: Instructions for the bodily warm-up and Body-Conversation Task (BCT)

S1.1. Original German version of instructions

EINLEITUNG

Wir kommen jetzt zu der Bewegungsaufgabe. Wir werden erst ein kurzes Warm-up machen, und dann eine Bewegungsaufgabe zu zweit. Am Anfang der beiden Bewegungsteile werde ich euch bitten eine "T-Pose" in Richtung Kameras zu machen und diese Pose zwei Sekunden zu halten (*Versuchsleiterin erklärt T-Pose*).

Denkt daran, dass es bei der Studie nicht um eine Leistungsbeurteilung geht. Ihr müsst euch nicht besonders schön oder viel oder intensiv bewegen. Ihr solltet euch immer in der Weise bewegen, die sich für euch selbst gerade passend anfühlt. Das kann manchmal auch sehr wenig sein oder sehr einfach sein. Übertreibt nicht und vor allem verletzt euch nicht. Es gibt kein richtig oder falsch, ihr könnt es einfach genießen und Spaß haben.

KÖRPERLICHE AUFWÄRMUNG

Bitte geht ihr in den Bereich, der mit dem Klebeband auf dem Boden markiert ist. Jeder von euch kann einen der beiden markierten Bereiche benutzen. Wir machen jetzt eine kurze körperliche Aufwärmung. Dazu führe ich euch in den ersten fünf Minuten durch einige einfache Bewegungsübungen. Ich möchte euch bitten, während der Aufwärmung nicht zu sprechen, es sei denn euch ist unwohl oder ihr benötigt Hilfe.

Versuchsleiterin schaltet die Kamera ein.

Wendet euch bitte in die Richtung der Kamera und macht die "T- Pose". Sehr gut. Jetzt kehrt bitte der anderen Person den Rücken und wendet euch jeweils in die Richtung der Tür und der Fenster. Genau so. Richtig. Erkundet ein wenig euren Bereich indem ihr ein bisschen in ihm herumläuft, bleibt dabei aber immer mit dem Rücken zur anderen Person. Ihr könnt zum Beispiel vorwärts und seitwärts laufen, große und kleine Schritte machen. *(Pause)* Ok. Kommt jetzt zur Mitte eures Bereichs. Ihr werdet euch jetzt ein bisschen auf der Stelle bewegen. Wir fangen bei den Füßen an. Lasst die Fußgelenke kreisen, erst einen Fuß, und dann den anderen. *(Pause)* Ok. Bewegt jetzt auch die ganzen Beine, die Füße und Fußgelenke, und auch eure Knie. Entdeckt einfach für einen Moment welche Bewegungen mit euren Beinen möglich sind. Ok. *(Pause)*

Geht jetzt bis zu den Hüften, ihr könnt die Hüften vor und zurück bewegen. *(Pause)* Oder auch zu den Seiten. *(Pause)* Wenn ihr möchtet könnt ihr sie kreisen lassen. *(Pause)* Sehr gut! Jetzt bewegt ihr auch eure Schultern und den gesamten Oberkörper: was immer sich dabei ok anfühlt. Ihr könnt zum Beispiel die Schultern, hoch und runter bewegen, *(Pause)* oder kreisen. *(Pause)* Ihr könnt den Oberkörper zum Beispiel nach rechts und links drehen, *(Pause)* oder hoch und runter gehen. Entdeckt wieder kurz für euch, was für Bewegungen mit eurem Oberkörper möglich sind. *(Pause)* Sehr gut. Beginnt jetzt, eure Arme zu kreisen. Eure ganze Arme, im großen Bogen. *(Pause)* Ok. Ihr könnt jetzt eure Arme frei bewegen und auch hier entdecken, welche Bewegungen ihr mit euren Armen machen könnt. *(Pause)* Bewegt dabei wenn ihr wollt auch eure Hände, lasst sie z.B. kreisen. *(Pause)* Sehr gut. Kommen wir jetzt noch zum Kopf. Bewegt euren Kopf langsam. Vor und zurück. *(Pause)* Und jetzt nach links und rechts. *(Pause)* Sehr gut. Wenn ihr möchtet könnt ihr den Kopf vorsichtig kreisen. *(Pause)*

Schaut jetzt nach oben und streckt euren ganzen Körper, so als würdet ihr Äpfel von einem Baum pflücken. Streckt euch schön nach oben und macht euch ganz groß. *(Pause)* Gut. Und jetzt gehet in die Knie nach unten und macht euch einmal ganz klein. *(Pause)* Sehr schön, ihr könnt wieder zum Stehen kommen. Nehmt euch jetzt einen Moment Zeit, um euren ganzen Körper zu bewegen, einfach so, wie ihr Lust habt. Versucht dabei immer noch der anderen Person den Rücken zu zukehren. Improvisiert ein bisschen mit eurer Bewegung. Einfach in eurem Tempo, und auf eurer eigene Art und Weise. *(Pause, ca. 15 sec.*)

Sehr gut. Zum Ende schüttelt jetzt nochmal alles ab. Schüttelt die Beine, die Arme, den Oberkörper. Ihr könnt gerne den Oberkörper hängen lassen und alles abschütteln. Sehr gut. Ihr könnt euch jetzt wieder zu mir drehen.

Versuchsleiterin schaltet die Kamera aus.

BODY-CONVERSATION TASK

Wir machen jetzt eine Bewegungsaufgabe zur zweit. Die Aufgabe ist folgende: Improvisiert oder erfindet gemeinsam Bewegungen, durch die ihr in Kontakt mit der anderen Person treten könnt. Versucht, euch durch Bewegung auszudrücken und versucht, durch Bewegung auf die andere Person zu reagieren und euch mit ihr in Beziehung zu treten. Stellt euch vor, dass ihr mit dieser Person kommunizieren wollt, aber ihr habt keine Worte, ihr habt nur den Körper und könnt nur durch Bewegung kommunizieren.

Versucht die ganze Zeit in Bewegung zu bleiben. Versucht dabei, euren ganzen Körper zu benutzen. Ihr könnt euch einfach frei bewegen und mit den Bewegungen spielen. Ihr könnt euch in eurem ganzen Bereich bewegen, aber bitte geht nicht außerhalb des Bereichs. Ihr könnt euch nach oben und nach unten bewegen, aber bitte setzt euch nicht hin und legt euch nicht hin. Und bitte berührt die andere Person nicht.

Ok. Habt ihr Fragen? (*Versuchsleiterin antwortet die Fragen, ohne zusätzliche Informationen zu geben*)

Die Interaktion wird 5 Minuten dauern und ich werde euch sagen, wann sie endet. Ich werde euch aber nicht zuschauen. Ich bleibe hier im Zimmer, falls ihr mich braucht, und drehe meinen Stuhl zur Wand. Wenn eine von euch die Interaktion aus irgendwelchen Gründen unterbrechen will, ihr könnt einfach "Stop" sagen und ich werde es abbrechen.

Versuchsleiterin dreht den Stuhl zur Wand hin, schaltet die Kameras ein und gibt folgende Anweisungen zum Starten:

Bitte macht die "T-Pose" in Richtung Kamera. Ok. Ich wiederhole jetzt die Aufgabe: improvisiert Bewegung gemeinsam mit der anderen Person. Stellt euch vor, ihr unterhaltet euch ohne Worte, nur durch Bewegung.

Nach 5 Minuten Versuchsleiterin sagt "Stop" und schaltet die Kamera aus.

S1.2. Translated English version of instructions

INTRODUCTION

We now come to the movement task. We will first do a short bodily warm-up and then a movement task in pairs. At the beginning of the two movement parts I will ask you to do a "T-Pose" towards the camera and to hold this pose for two seconds (*Experimenter clarifies the T-Pose*).

Remember that this study is not about an assessment of performance. You don't have to move very nicely, very much or intensively. You should always move in the way that feels right for you. This can sometimes be very little or very simple. Don't exaggerate and above all don't hurt yourself. There is no right or wrong, you can just enjoy it and have fun.

BODILY WARM-UP

Please go to the area marked with the tape on the floor. Each of you can use one of the two marked areas. We will now do a short bodily warm-up. In these first five minutes I will guide you through some simple movement exercises. I would like to ask you not to speak during the warm-up unless you feel uncomfortable or need help.

Experimenter turns on the cameras

Please turn towards the camera and do the "T- Pose". Very good. Now please turn your back to the other person and turn respectively towards the door and the windows. Just like that. That's right. Explore your area a bit by walking around in it, but always keep your back to the other person. For example, you can walk forward and sideways, you can make big and small steps. *(Pause)* Ok. Now come to the center of your area. You will now move a little on the spot. Let's start with the feet. Let the ankles circle, first one foot, and then the other. *(Pause)* Ok. Now move all your legs, together with the feet and ankles, and also your knees. Just discover for a moment which movements are possible with your legs. *(Pause)*

Ok. Now move the hips, you can move the hips back and forth. *(Pause)* Or also to the sides. *(Pause)* If you want you can also circle them. *(Pause)* Very good. Now move also your shoulders and the whole upper body: whatever feels okay. For example, you can move your shoulders up and down, *(Pause)* or circle them. *(Pause)* You can for example turn your upper body to the right and to the left, *(Pause)* or go up and down. Discover again briefly for yourself what kind of movements are possible with your upper body. *(Pause)* Very good. Now begin to circle your arms. All your arms, in big arcs. *(Pause)* Ok. You can now move your arms freely and again explore which movements you can make with your arms. *(Pause)* Move your hands if you want to, let them circle for example. *(Pause)* Very good. Now let's get to the head. Move your head slowly. Back and forth. *(Pause)* And now to the left and to the right. *(Pause)* Very good. If you want, you can circle your head carefully. *(Pause)*

Now look up and stretch your whole body as if you were picking apples from a tree. Stretch up nicely and make yourself big. *(Pause)* Good. And now bend your knees and make yourselves very small. *(Pause)* Very nice, you can now come to standing. Now take a moment to move your whole body just as you like. Try to keep turning your back to the other person. Improvise a little with your movement. Simply at your pace, and in your own way. *(Pause, approx. 15 sec.)*

Very good. At the end shake everything off. Shake your legs, arms, and upper body. If you like, you can let your upper body hang and shake everything off. *(Pause)* Very good. Now you can turn to me.

Experimenter turns off the cameras

BODY-CONVERSATION TASK

We now do a movement task in pairs. The task is the following: improvise movement together and try to get in contact with the other person. Try to express yourself through movement and try to react and relate to the other person through movement. Imagine that you want to communicate with this person but you have no words, you have only the body and you can only communicate through movement.

Try to keep moving all the time. Try to use your whole body. You can move freely and play with the movements. You can move within your designated area but please don't go out of this space. You can move upwards and downwards, but please don't sit nor lie down. And please do not touch the other person.

Ok. Do you have any questions? *(When answering, the experimenter clarifies what is already stated in the instructions and avoids giving additional information)*

The interaction will last five minutes and I will tell you when it ends. But I am not going to watch you. I will stay in the room, in case you need me, and turn my chair to the wall. If one of you wants to stop the interaction for any reason, you can just say “stop” and I will interrupt the interaction.

Experimenter turns her chair to the wall, turns on the cameras, and gives the following instructions to begin:

Please do the “T pose” towards the camera. Ok. I now repeat the task: Improvise movement together with the other person. Imagine you are having a conversation without words, only through movement.

After 5 minutes Experimenter says "stop" and turns off the cameras.
